# Association of Cardiac Troponin T With Coronary Atherosclerosis in Asymptomatic Primary Prevention People With HIV

**DOI:** 10.1016/j.jacadv.2024.101206

**Published:** 2024-08-16

**Authors:** Christopher deFilippi, Sara McCallum, Markella V. Zanni, Kathleen V. Fitch, Marissa R. Diggs, Gerald S. Bloomfield, Carl J. Fichtenbaum, Judith A. Aberg, Carlos D. Malvestutto, Adriana Pinto-Martinez, Ann Stapleton, Joan Duggan, Gregory K. Robbins, Jana Taron, Julia Karady, Borek Foldyna, Michael T. Lu, Heather J. Ribaudo, Pamela S. Douglas, Steven K. Grinspoon

**Affiliations:** a Inova Heart and Vascular Institute, Falls Church, Virginia, USA; b Metabolism Unit, Massachusetts General Hospital and Harvard Medical School, Boston, Massachusetts, USA; c Department of Medicine, Duke Global Health Institute and Duke Clinical Research Institute, Duke University, Durham, North Carolina, USA; d Division of Infectious Diseases, University of Cincinnati College of Medicine, Cincinnati, Ohio, USA; e Division of Infectious Diseases, Icahn School of Medicine at Mount Sinai, New York, New York, USA; f Division of Infectious Diseases, Ohio State University Medical Center, Columbus, Ohio, USA; g HIV Unit, Hospital Universitario 12 De Octubre – Imas12, Madrid, Spain; h Eisenhower Health Center at Rimrock, Eisenhower Health, Rancho Mirage, California, USA; i Division of Infectious Diseases, University of Toledo, Toledo, Ohio, USA; j Division of Infectious Diseases, Massachusetts General Hospital and Harvard Medical School, Boston, Massachusetts, USA; k Department of Radiology, Faculty of Medicine, Medical Center – University of Freiburg, University of Freiburg, Freiburg, Germany; l Cardiovascular Imaging Research Center, Department of Radiology, Massachusetts General Hospital and Harvard Medical School, Boston, Massachusetts, USA; m Cardiovascular Imaging Research Group, Heart and Vascular Center, Semmelweis University, Budapest, Hungary; n Center for Biostatistics in AIDS Research, Harvard T.H. Chan School of Public Health, Boston, Massachusetts, USA; o Duke University Research Institute, Duke University School of Medicine, Durham, North Carolina, USA

**Keywords:** coronary artery disease, troponin, HIV, coronary computed tomography angiography, predictive modeling

## Abstract

**Background:**

Coronary plaque is common among people with HIV (PWH) with low-to-moderate traditional atherosclerotic cardiovascular disease (ASCVD) risk.

**Objectives:**

The purpose of this study was to determine the association of high-sensitivity cardiac troponin T (hs-cTnT) levels with coronary plaque characteristics and evaluate if hs-cTnT improves identification of these features beyond traditional ASCVD risk factors among PWH.

**Methods:**

Among PWH receiving stable antiretroviral therapy with low-to-moderate ASCVD risk and no known history of ASCVD, hs-cTnT levels and measures of plaque by coronary computed tomography angiography were assessed. Primary outcomes included the association of hs-cTnT level with the presence of any plaque, vulnerable plaque, coronary artery calcium (CAC) score, and Leaman score. Assessment of model discrimination of hs-cTnT for plaque characteristics was also performed.

**Results:**

The cohort included 708 U.S. participants with a mean age of 51 ± 6 years, 119 (17%) females, a median ASCVD risk score of 4.4% (Q1-Q3: 2.5%-6.6%), and a median hs-cTnT level of 6.7 ng/L (detectable level ≥6 ng/L in 61%). Any plaque was present in 341 (48%), vulnerable plaque in 155 (22%), CAC>100 in 68 (10%), and a Leaman score >5 in 105 (15%). After adjustment for ASCVD risk score, participants with hs-cTnT >9.6 ng/L (highest category) versus an undetectable level (<6 ng/L) had a greater relative risk for any plaque (1.37, 95% CI: 1.12-1.67), vulnerable plaque (1.47, 95% CI: 1.16-1.87), CAC>100 (2.58, 95% CI: 1.37-4.83), and Leaman score >5 (2.13, 95% CI: 1.32-3.46). The addition of hs-cTnT level modestly improved the discrimination of ASCVD risk score to identify critical plaque features.

**Conclusions:**

In PWH without known ASCVD, hs-cTnT levels were strongly associated with and improved prediction of subclinical coronary plaque. (Evaluating the Use of Pitavastatin to Reduce the Risk of Cardiovascular Disease in HIV-Infected Adults [REPRIEVE]; NCT02344290)

People with HIV (PWH) are at an increased risk of cardiovascular disease (CVD) despite effective antiretroviral therapy (ART).^1^ The REPRIEVE (Randomized Trial to Prevent Vascular Events in HIV) identified that a statin strategy prevented major adverse cardiovascular events (MACE) among PWH without known CVD, on ART, with low-to-moderate traditional risk.^2^^,^^3^ Baseline data from the REPRIEVE mechanistic substudy employing coronary computed tomography angiography (CCTA) revealed that 47% of PWH participants with a median 10-year pooled cohort atherosclerotic cardiovascular disease (ASCVD) risk score of <5.0% had measurable coronary plaque,^4^ highlighting the need for complementary strategies to predict subclinical plaque and ensuing ASCVD risk.^5^ Circulating biomarkers may augment ASCVD risk assessment among PWH with only low-to-moderate traditional ASCVD risk. High-sensitivity cardiac troponin T (hs-cTnT) is cardiac-specific, clinically available, and a powerful prognosticator of CVD events in the general population without acute coronary syndromes (ACS).^6^ Furthermore, hs-cTnI and T have been associated with higher-risk coronary plaque characteristics in people with chest pain in the absence of an ACS event or HIV.^7^, ^8^, ^9^ In people without HIV, high-risk plaque characteristics are associated with an increased risk of MACE.^10^, ^11^, ^12^ To further identify useful biomarkers for plaque and potentially for MACE in a primary prevention strategy, we leveraged the mechanistic substudy of REPRIEVE to determine the prevalence of elevated hs-cTnT, the association of hs-cTnT with coronary plaque and high-risk plaque characteristics, and the utility of hs-cTnT to augment discrimination of plaque features by traditional risk scoring.

## Methods

### Study participants

Participants are ART-treated PWH between the ages of 40 to 75 years, without known CVD, with a low-to-moderate traditional ASCVD risk participating in REPRIEVE (NCT02344290) and the mechanistic CCTA substudy.^5^^,^^13^ Further details regarding inclusion and exclusion into REPRIEVE are provided elsewhere.^13^^,^^14^ The larger main REPRIEVE trial population consisted of 7,769 participants of which 755 participants from 31 U.S. REPRIEVE sites participated in the CCTA substudy.^2^^,^^4^ The clinical characteristics of the substudy participants were overall similar to the total U.S. REPRIEVE participants (n = 3,788) except for a lower proportion of women and a higher proportion of Whites.^4^ Each clinical research site obtained Institutional Review Board/ethics committee approval. Participants signed the approved informed consent.

### Hs-cTnT measurement

Hs-cTnT was collected at enrollment and measured on the Cobas e602 (Roche Diagnostics) at the Inova Biocore laboratory using previously frozen plasma stored at −80 ^°^C that had undergone one prior freeze-thaw cycle. We used a fifth-generation assay clinically available in the United States. This assay has a lower limit of detection of 6 ng/L.^15^

### Coronary CTA

Baseline image acquisition was performed between 2015 and 2018, prior to treatment initiation. Images were interpreted by the REPRIEVE CT core laboratory for the presence, extent, and composition of coronary plaque as previously reported.^4^

### Outcomes

We evaluated the associations of hs-cTnT levels with the presence of any plaque, vulnerable plaque characteristics (including low attenuation, napkin ring sign, and positive remodeling), coronary artery calcium (CAC) score, and Leaman score (which quantifies the overall burden of coronary artery disease considering degree of stenosis, coronary dominance, plaque location, and composition).^16^

### Statistics

Continuous outcomes are presented as mean ± SD or median (Q1-Q3). Categorical outcomes are shown as an absolute number (percent). Hs-cTnT is presented as undetectable (<6 ng/L) and by tertiles of quantifiable values to give four roughly equal ordered categories. For demographics and baseline characteristics, associations with hs-cTnT were performed using Spearman’s Ρ (rho) for continuous variables, the Cochran–Mantel–Haenszel test with rank-scores for ordinal variables, and the Jonckheere-Terpestre test for nonbinary categorical variables. For the plaque outcomes (prevalence of plaque feature), trends across hs-cTnT categories were tested using the Cochran-Armitage test for trend for the binary plaque outcomes, and linear-by-linear association for nonbinary categorical plaque outcomes. Using log-binomial regression, the association of hs-cTnT (ordinal exposure) to plaque and its components (outcomes) was evaluated unadjusted, adjusted for the 10-year ASCVD score (as a nonbinary categorical variable [<2.5%, 2.5%-<5%, ≥5%] consistent categories previously used in the REPRIEVE CCTA substudy collapsing the >10% category due to very few participants), and further for biomarkers of inflammation previously shown to be associated with plaque outcome including C-reactive protein, interleukin-6, monocyte chemoattractant protein-1 (MCP-1), lipoprotein-associated phospholipase A2 (Lp-PLA-2), and oxidized low-density lipoprotein (LDL) all modeled as continuous variables, and nadir CD4 (modeled as a nonbinary categorical outcome as collected).^4^ We performed a sensitivity analysis using logistic regression modeling. In supplemental unadjusted analyses, we evaluated the association of hs-cTnT to plaque with measurable hs-cTnT level included as continuous variable using natural cubic splines with 3 and with 5 knots. The area under the receiver-operating characteristic curve was calculated for each plaque characteristic with the c-index for the ASCVD risk score (categorical measure) and then the increment in area under the curve (AUC) with the addition of hs-cTnT level (four categories as described above). A *P* value was determined for the incremental AUC increase over ASCVD risk score alone. Analyses used SAS software, version 9.4M7 on a Linux platform.

## Results

Of 755 participants with complete CCTA available for interpretation, 708 had plasma available for hs-cTnT measurement. Supplemental Table 1 shows baseline clinical, HIV, and CCTA characteristics by category of hs-cTnT level. Participants had a mean age of 51 ± 6 years, were 119 (17%) females, 440 (62%) non-White, with a median ASCVD risk score of 4.4 (Q1-Q3: 2.5-6.6), and a median hs-cTnT level of 6.7 ng/L, detectable (≥6 ng/L) in 61%. Age, ASCVD risk score, systolic blood pressure, and proportion of male sex all increased across progressive categories of hs-cTnT. HIV-specific factors were not associated with hs-cTnT levels (Supplemental Table 1).

**Figure 1:**
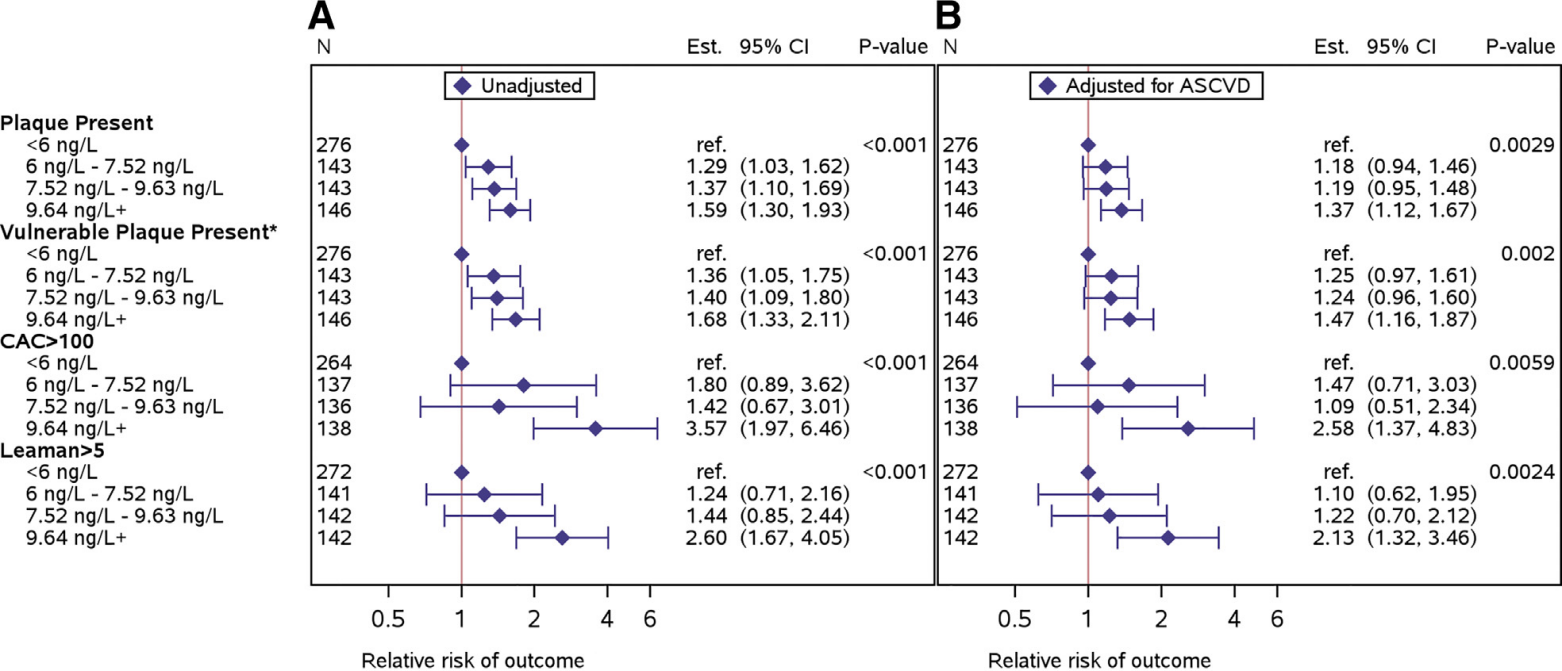
**Log Binomial Regression on Binary Plaque Outcomes by hs-cTnT Level for the Pooled Cohort 10-Year ASCVD Risk Score** (A) Unadjusted and (B) adjusted. ∗Plaque with Visible Noncalcified Portion or Plaque with Vulnerable Features. The *P*-value tests for a linearly increasing log RR. ASCVD = atherosclerotic cardiovascular disease; CAC = coronary artery calcium; hs-cTnT = high-sensitivity cardiac troponin.

**Figure 2:**
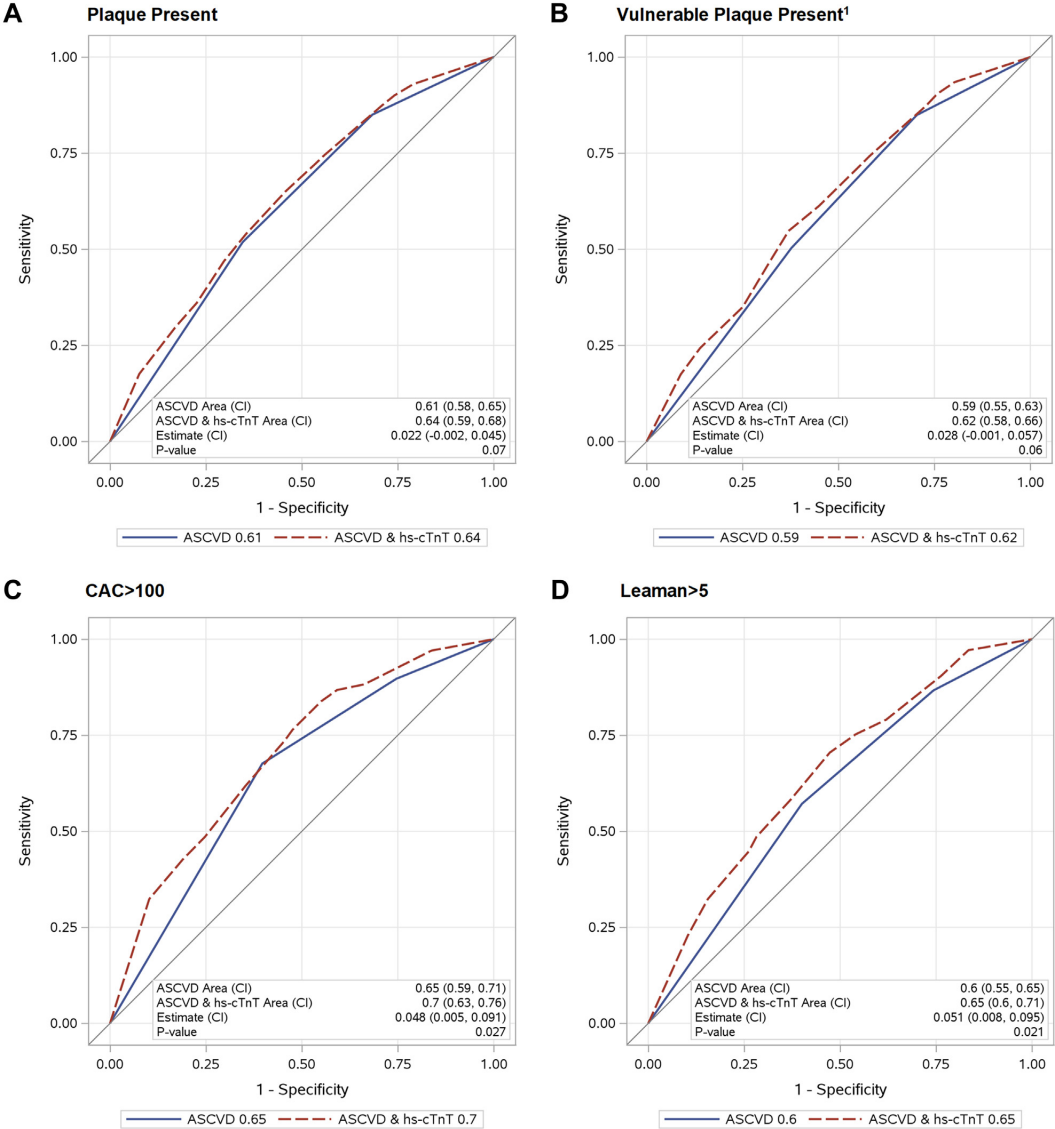
**Area Under the Curve for Binary Plaque Outcomes, for ASCVD Risk Score, and the Addition of hs-cTnT Level** Assessment of AUC for (A) Presence of Plaque; (B) Presence of Vulnerable Plaque1; (C) CAC Score >100; (D) Leaman Score >5. ^1^Plaque with Visible Noncalcified Portion or Plaque with Vulnerable Features. Significant *P*-values indicate that the addition of hs-cTnT to the model provides improved prediction over and above the reference alone (ASCVD risk score). Abbreviations as in Figure 1.

For CCTA findings, progressive hs-cTnT categories were associated with a higher prevalence of all plaque characteristics including the presence of any coronary plaque, and higher-risk phenotypes including plaque with vulnerable characteristics, CAC score >100, and Leaman score >5 (Table 1). The distribution of hs-cTnT levels based on these 4 CCTA characteristics is presented in Supplemental Figure 1. Forest plots for progressive hs-cTnT categories for each of the four CCTA characteristics are shown in unadjusted and adjusted log binomial regression analysis in Figure 1. In a supplemental analysis, we evaluated measurable hs-cTnT as a continuous variable using natural cubic splines that also showed that progressively higher measurable hs-cTnT levels are associated with a higher prevalence of the 4-plaque features (Supplemental Figure 2). Therefore, we utilized the planned categorical assessment of hs-cTnT throughout the remainder of the analysis. After adjustment for baseline ASCVD score, the participants with the highest category of hs-cTnT (≥9.6 ng/L) had a 37% higher prevalence of any plaque, a 47% higher prevalence of vulnerable plaque, a 158% higher prevalence of a CAC score >100, and a 113% greater prevalence of a Leaman score >5, versus participants with a hs-cTnT level <6 ng/L. Intermediate levels of hs-cTnT had intermediate levels of association with increased risk for vulnerable plaque characteristics but did not achieve statistical significance after adjustment. Additional adjustment for biomarkers of inflammation and nadir CD4 counts minimally changed the point estimates (Supplemental Figure 3). Similar results were seen in logistic regression modeling (Supplemental Figure 4). Furthermore, the addition of hs-cTnT levels to the 10-year ASCVD risk score improved model discrimination for identifying all 4 plaque characteristics with higher CAC and Leaman score achieving the greatest change in AUC of 0.05 for both (Figure 2).

**Table 1:** Plaque Outcomes by hs-cTnT Level

		All Participants					
		Total (N = 708)	<6 ng/L (n = 276)	6-7.52 ng/L (n = 143)	7.52-9.63 ng/L (n = 143)	9.64 ng/L+ (n = 146)	*P* Value
Participants with plaque present^a^	Yes	341 (48%)	106 (38%)	71 (50%)	75 (52%)	89 (61%)	**<0.0001**
Plaque with visible noncalcified portion^a^	Yes	277 (39%)	82 (30%)	59 (41%)	62 (43%)	74 (51%)	**<0.0001**
Number of visible noncalcified segments^b^	0	431 (61%)	194 (70%)	84 (59%)	81 (57%)	72 (49%)	**<0.0001**
	1-2	226 (32%)	67 (24%)	50 (35%)	52 (36%)	57 (39%)	-
	>=3	51 (7%)	15 (5%)	9 (6%)	10 (7%)	17 (12%)	-
Plaque with vulnerable features^a^	Yes	155 (22%)	41 (15%)	33 (23%)	37 (26%)	44 (30%)	**0.0001**
Low attenuation plaque	Yes	41 (6%)	9 (3%)	8 (6%)	9 (6%)	15 (10%)	-
Napkin ring sign	Yes	21 (3%)	8 (3%)	3 (2%)	3 (2%)	7 (5%)	-
Positive remodeling	Yes	150 (21%)	40 (14%)	31 (22%)	36 (25%)	43 (29%)	-
Plaque with noncalcified portion or plaque with vulnerable features^a^	Yes	292 (41%)	88 (32%)	62 (43%)	64 (45%)	78 (53%)	**<0.0001**
Calcium score (Agatston)^a^	>0	234 (35%)	75 (28%)	52 (38%)	45 (33%)	62 (45%)	**0.003**
	*# missing*	*33*	*12*	*6*	*7*	*8*	*-*
Among those with CAC > 100	1-100	166 (71%)	60 (80%)	38 (73%)	34 (76%)	34 (55%)	0.150
	101-400	56 (24%)	11 (15%)	11 (21%)	10 (22%)	24 (39%)	-
	>400	12 (5%)	4 (5%)	3 (6%)	1 (2%)	4 (6%)	-
Calcium score (Agatston) (all groups)^b^	0	441 (65%)	189 (72%)	85 (62%)	91 (67%)	76 (55%)	**0.0002**
	1-100	166 (25%)	60 (23%)	38 (28%)	34 (25%)	34 (25%)	-
	101-400	56 (8%)	11 (4%)	11 (8%)	10 (7%)	24 (17%)	-
	>400	12 (2%)	4 (2%)	3 (2%)	1 (1%)	4 (3%)	-
Leaman score category^b^	0	367 (53%)	170 (63%)	72 (51%)	68 (48%)	57 (40%)	**<0.0001**
	>0-5	225 (32%)	74 (27%)	51 (36%)	53 (37%)	47 (33%)	-
	>5	105 (15%)	28 (10%)	18 (13%)	21 (15%)	38 (27%)	-
Maximum stenosis^b^	0%	367 (53%)	170 (63%)	72 (51%)	68 (48%)	57 (40%)	**<0.0001**
	1%-49%	308 (44%)	95 (35%)	68 (48%)	71 (50%)	74 (52%)	-
	50%-69%	15 (2%)	3 (1%)	1 (1%)	3 (2%)	8 (6%)	-
	70%-100% or ≥50% left main	7 (1%)	4 (1%)	0 (0%)	0 (0%)	3 (2%)	-
	*# missing*	*11*	*4*	*2*	*1*	*4*	*-*

**Bold** text indicates significant *P* values.

CAC = coronary artery calcium; hs-cTnT = high-sensitivity cardiac troponin.

a Cochran-Armitage trend test.

b Linear-by-linear association test.

## Discussion

The REPRIEVE trial recently showed the efficacy of a primary statin intervention to prevent MACE in low-moderate risk, PWH.^2^ Despite the success of REPRIEVE, critical questions remain as to how best to identify a primary prevention population in PWH, and whether circulating markers associated with plaque may be useful in this regard. Within REPRIEVE, a large embedded mechanistic study assessed coronary plaque and biomarkers showing significant reductions in plaque.^17^ In addition, baseline plaque data from REPRIEVE has shown a surprisingly high prevalence of high-risk plaque.^4^ In the current study, we add to these findings assessing how well hs-cTnT levels discriminate plaque. Among a large group of well defined, asymptomatic PWH, we show higher levels of hs-cTnT, a biomarker specific for cardiomyocyte death or injury, related to the presence of coronary plaque and higher-risk plaque characteristics (Central Illustration). Traditional ASCVD risk scores underestimate the risk of incident CVD events in PWH.^18^ Here we show that hs-cTnT improved model discrimination, beyond that achieved with ASCVD risk scoring, for higher-risk coronary plaque imaging features that are most likely to progress to an ASCVD adverse event.^5^^,^^19^ These data advance the field and suggest the need to determine whether hs-cTnT can be used as a predictor of MACE, statin effects on MACE, and statin effects on plaque in future studies of PWH.

**Central Illustration:**
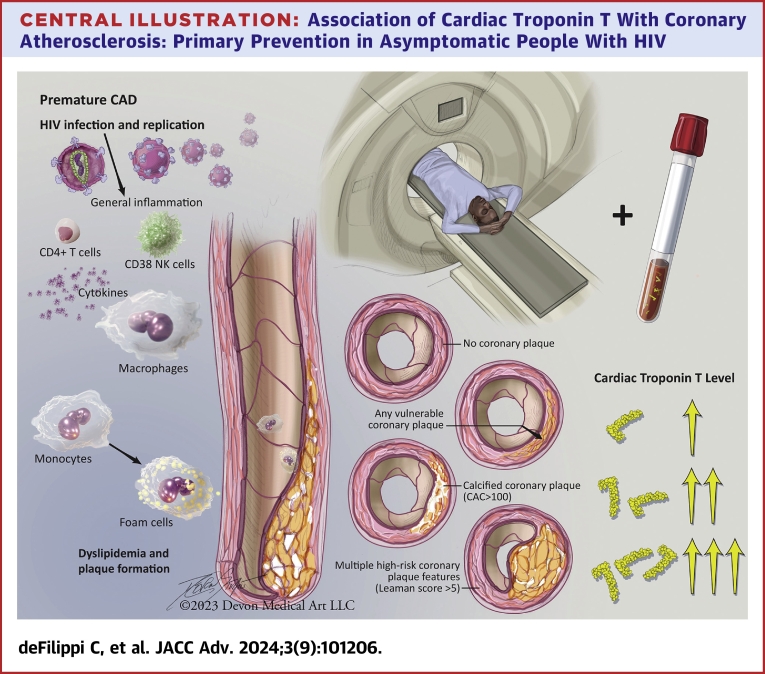
**Association of Cardiac Troponin T With Coronary Atherosclerosis: Primary Prevention in Asymptomatic People With HIV** People with HIV (PWH) have an increased prevalence of coronary atherosclerosis including higher risk plaque features measured by coronary CT angiography (CCTA). Levels of circulating cardiac troponin T measured with a high sensitivity assay are independently associated with the prevalence of coronary plaque, particularly with plaque features associated with higher risk for major adverse cardiac events. Abbreviation as in Figure 1.

Prior studies from non-HIV general population and chest pain without ACS cohorts have consistently demonstrated an association between vulnerable plaque morphology features by CCTA and longer-term risk of MACE (Supplemental Table 2).^10^, ^11^, ^12^ Furthermore, CCTA studies of populations of patients without HIV and ACS have consistently found independent associations between high-risk coronary plaque features and progressively higher measures of hs-cTnI or T well below the 99th percentile upper reference limit identified for cTn recommended as a cutoff for acute MI diagnosis as part of the 4th Universal Definition of Myocardial Infarction.^7^, ^8^, ^9^^,^^20^^,^^21^ In contrast, despite evidence of increased high-risk plaque in PWH, limited data are available investigating the utility of hs-cTnI or T among PWH, particularly in the large virologically controlled group of PWH being considered for primary cardiovascular prevention.

Three prior studies measured hs-cTnI or T in PWH without known CVD undergoing CCTA and reported discordant results. In a pilot study of 58 PWH, hs-cTnT levels were associated with noncalcified plaque in a model accounting for CVD- and HIV-specific risk factors.^22^ In addition to a small sample size limiting adjustment for confounders, this population of PWH had a particularly high prevalence, of nearly 90%, with respect to cocaine and tobacco use. Both could influence coronary plaque extent and morphology as well as, potentially hs-cTnT levels. This limits the generalizability of this study’s results to more heterogeneous PWH populations including REPRIEVE where tobacco use was nearly four-fold less. In another study of 155 PWH, hs-cTnT was associated with any plaque and coronary calcium, but not noncalcified plaque.^23^ The directionality of the effect estimates was similar to our findings within the CCTA sub study of REPRIEVE with hs-cTnT showing a greater relative risk for CAC score>100 than the presence of vulnerable plaque.^23^ In contrast, in a substudy of the MACS (Multicenter AIDS Cohort Study) assessing 458 at-risk males, 272 had HIV (59% of the MACS sub-study cohort). Among this group, hs-cTnI was associated only with obstructive CAD and no other plaque characteristics.^24^ Notably, beyond a smaller sample size that may have underpowered the study to detect more differences in plaque morphology based on hs-cTnI levels, MACS PWH were different from the REPRIEVE mechanistic cohort with >80% of the MACS PWH having detectable HIV RNA and only 61% taking ART, increasing the probability of non-plaque-related etiologies contributing to myocyte injury.^25^ Furthermore, technical issues, such as the use of earlier generation CT scanners with lower resolution and potentially the choice of hs-cTnI versus hs-cTnT could all account in part for differences between the MACS and REPRIEVE CCTA findings.

PWH living with constant low-level inflammation despite viral suppression may have competing mechanisms for chronic cTn release into the circulation from myocytes that are both ischemic and nonischemic in etiology.^25^, ^26^, ^27^ In this regard, the presence of detectable troponin in 61% is striking, given the absence of any clinical history of ASCVD. Notably, our results linking troponin to plaque remained robust controlling for markers of inflammation, suggesting a strong independent association of hs-cTnT with plaque. This also needs to be put in context that cTnT may be expressed in skeletal muscle in chronic disease states impacting skeletal muscle and even with just advanced age.^28^^,^^29^ Our data from a large, asymptomatic primary prevention population of PWH, well controlled on ART and with low predicted ASCVD risk, significantly extend our knowledge of the potential utility of hs-cTnT as an independent marker of plaque. This observation is important and has clinical relevance, as prior studies have failed to identify robust predictors of plaque in a well-defined primary prevention population of PWH. Indeed, the findings from REPRIEVE, a population with low to moderate predicted risk, are consistent with findings in high-risk non-HIV populations, linking hs-cTnI or T to vulnerable plaque.^7^, ^8^, ^9^ The use of hs-cTnT levels to augment identification of high-risk subclinical coronary disease needs to be put in clinical context. For reference, single measurements of hs-cTnT for the diagnosis of myocardial infarction in a large general population chest pain cohort has a sensitivity of 89.9% and a specificity of 79.8%, with an AUCs reported as 0.93.^5^^,^^30^ In the general population, an ASCVD 10-year risk score ≥7.5% is associated with a recommendation to consider measuring a CAC score and starting a statin for a score ≥100.^5^ The incremental increase in association of high-risk subclinical coronary disease when hs-cTnT level is added to the ASCVD risk-score can motivate the assessment of this biomarker to test if it can augment incident ASCVD risk prediction in the full clinical cohort.

This study is the largest among PWH, providing novel data on the relationship of hs-cTnT to plaque in PWH, but has some limitations. The study is cross-sectional, and analyses will be performed to assess hs-cTnT longitudinally in relationship to plaque changes and MACE in REPRIEVE. Higher-risk plaque characteristics are associated with progressively higher hs-cTnT levels, but other potential mechanisms will need to be further investigated. Our findings are from participants selected for a primary cardiovascular prevention trial, representative of the large population of well-controlled PWH on ART in the United States, but generalization to other groups of PWH in other Global Burden of Disease regions and with varying degrees of ART use and virologic suppression will need to be determined.

## Conclusions

hs-cTnT levels are strongly associated with coronary plaque and higher-risk plaque characteristics, providing useful information beyond the 10-year ASCVD risk score to identify coronary atherosclerosis among asymptomatic PWH at low-to-moderate traditional ASCVD risk. Further investigation will determine whether hs-cTnT levels may help identify individuals with HIV who are more likely to develop incident ASCVD and who would benefit from primary prevention treatment strategies.

Perspectives **COMPETENCY IN MEDICAL KNOWLEDGE:** To what extent do levels of the circulating biomarker cardiac troponin T, a measure of myocyte injury and cell death, associate with presence of subclinical coronary plaque and high-risk plaque characteristics in PWH who are asymptomatic, without known ASCVD, and low-to-moderate traditional risk.

**TRANSLATIONAL OUTLOOK:** In this cross-sectional study of 708 participants with well-controlled HIV undergoing CCTA, progressively higher subclinical elevations in cardiac troponin T levels as measured with high-sensitivity troponin assay were strongly associated with the presence of coronary artery plaque and higher-risk plaque characteristics, after adjusting for traditional cardiovascular risk factors, HIV-specific factors, and circulating markers of inflammation. Cardiac troponin T levels may help to identify higher-risk coronary plaque characteristics among PWH without known cardiovascular disease.

## Funding support and author disclosures

The views expressed in this manuscript are those of the authors and do not necessarily represent the views of the National Heart, Lung, and Blood Institute or the National Institute of Allergy and Infectious Diseases; the National Institutes of Health; or the U.S. Department of Health and Human Services. This study is supported through NIH grants U01HL123336, to the Clinical Coordinating Center, and U01HL123339, to the Data Coordinating Center as well as funding from Kowa Pharmaceuticals America, Inc, Gilead Sciences, and ViiV Healthcare. The NIAID supported this study through grants UM1 AI068636, which supports the Advancing Clinical Therapeutics Globally (ACTG) Network Leadership and Operations Center; and UM1 AI106701, which supports the ACTG Laboratory Center. This work was also supported by the Nutrition Obesity Research Center at Harvard (P30DK040561 to SKG). Dr deFilippi has received grant support to his institution from Roche Diagnostics and consulting for Roche Diagnostics which manufacturers the troponin T assay; has received grants to his institution from Abbott Diagnostics, FujiRebio, Quidel/Ortho, Siemens Healthineers, and the NHLBI outside this submitted work; consulting for Abbott Diagnostics, FujiRebio, and Quidel/Ortho, and Siemens Healthineers; and serves as a member of the clinical endpoint committee for Siemens Healthineers. Dr Zanni is the principal investigator of research grants from 10.13039/100000002NIH (NIAID and NHLBI) and from 10.13039/100005564Gilead Sciences to her institution and participating in a DSMB for 10.13039/100000002NIH-funded studies involving no compensation. Dr Fichtenbaum has received grant support through his institution from 10.13039/100005564Gilead Sciences, 10.13039/100010877ViiV Healthcare, 10.13039/100004330GSK, 10.13039/100005565Janssen, 10.13039/100006483Abbvie, 10.13039/100004334Merck, 10.13039/100002429Amgen, and Cytodyn, outside the submitted work; and personal fees from Theratechnologies and ViiV for consulting and participation on Advisory Board unrelated to REPRIEVE with Theratechnologies and ViiV, and role as Chair on DSMB for Intrepid Study, outside the submitted work. Dr Aberg has received institutional research support for clinical trials from Emergent Biosolutions, Frontier Technologies, Gilead Sciences, GlaxoSmithKline, Janssen, MacroGenics, Merck, Pfizer, Regeneron, and ViiV Healthcare; and personal fees for advisory boards from Glaxo Smith Kline/Viiv and Merck and participation on DSMB for Kintor Pharmaceuticals, all outside the submitted work. Dr Malvestutto has received institutional research support by Lilly; and honoraria from 10.13039/100010877ViiV Healthcare and 10.13039/100005564Gilead Sciences for Advisory Board membership, outside the submitted work. Dr Pinto-Martinez has received honoraria from Gilead, Janssen, and ViiV Healthcare for presentations and educational events, outside the submitted work. Dr Stapleton has received institutional research support by 10.13039/100000002NIH. Dr Robbins has received grant support to his institution from Leonard-Meron Bioscience; consulting fees to his institution from Seed Inc and Teradyne Inc; payment for expert testimony from Tufts Medical Center, participation on a DSMB for an NIH trial, and unpaid membership on a review panel for DHHS OI Guidelines. Dr Taron has received support from 10.13039/501100001659Deutsche Forschungsgesellschaft (DFG, German Research Foundation) relevant to the present work; consulting fees from Universimed Cross Media Content GmbH, Core Lab Black Forrest GmbH; and payments or honoraria from Siemens Healthcare GmbH, Bayer AG, outside of the submitted work. Dr Foldyna has received institutional support from 10.13039/100004325AstraZeneca, 10.13039/501100004628MedImmune, and MedTrace, outside of the submitted work. Dr Lu has received grant support through his institution from Kowa Pharmaceuticals America, Inc, for the conduct of the study; grant support from 10.13039/501100004628MedImmune and 10.13039/100004325AstraZeneca; and personal fees from PQBypass, outside of the current work. Dr Ribaudo has received grants from 10.13039/100000002NIH/10.13039/100000050NHLBI and Kowa Pharmaceuticals during the conduct of the study as well as grants from 10.13039/100000002NIH/10.13039/100000060NIAID, and 10.13039/100000002NIH/10.13039/100000050NHLBI, 10.13039/100000002NIH/10.13039/100000062NIDDK, and 10.13039/100000002NIH/10.13039/100000049NIA outside the submitted work. Dr Grinspoon has received grant support through his institution from Kowa Pharmaceuticals America, Inc 10.13039/100005564Gilead Sciences, Inc; and 10.13039/100010877ViiV Healthcare for the conduct of the study, as well as grants from Theratechnologies and Navidea and personal fees from Theratechnologies and ViiV, all outside the submitted work; and is a member of the Scientific Advisory Board of Marathon Asset management. All other authors have reported that they have no relationships relevant to the contents of this paper to disclose.
