# Supplementary material for: Association of Cardiac Troponin T With Coronary Atherosclerosis in Asymptomatic Primary Prevention People With HIV
Source: JACC Adv. 2024 Aug 16;3(9):101206. doi: 10.1016/j.jacadv.2024.101206 (PMC11381782; doi:10.1016/j.jacadv.2024.101206)
Supplement: Supplemental Data [file mmc1.docx]

**Supplemental Table 1: Demographic Characteristics by hs-cTnT Level**

| **Characteristic** |  | **Total (N=708)** | **<6 ng/L (N=276)** | **6 ng/L - 7.52 ng/L (N=143)** | **7.52 ng/L - 9.63 ng/L (N=143)** | **9.64 ng/L+ (N=146)** | **P-value (rho*)** |
| --- | --- | --- | --- | --- | --- | --- | --- |
| ***Overall*** | | | | | | | |
| All participants |  | 708 | 276 (39%) | 143 (20%) | 143 (20%) | 146 (21%) |  |
|  | | | | | | | |
|  | | | | | | | |
| ***Demographics*** | | | | | | | |
| Age (years) | Mean (s.d.) | 50.8 (5.8) | 48.9 (5.1) | 50.7 (5.4) | 51.5 (5.7) | 53.6 (6.1) | <0.0001 (0.3) |
|  | | | | | | | |
| Natal sex | Female | 119 (17%) | 79 (29%) | 14 (10%) | 16 (11%) | 10 (7%) | <0.0001 |
|  | | | | | | | |
| Race/Ethnicity^2^ | White Non-Hispanic | 268 (38%) | 93 (34%) | 67 (47%) | 58 (41%) | 50 (34%) | 0.050 |
|  | Black Non-Hispanic | 242 (34%) | 87 (32%) | 38 (27%) | 50 (35%) | 67 (46%) |  |
|  | Hispanic | 171 (24%) | 86 (31%) | 31 (22%) | 30 (21%) | 24 (16%) |  |
|  | Other | 27 (4%) | 10 (4%) | 7 (5%) | 5 (3%) | 5 (3%) |  |
|  | | | | | | | |
|  | | | | | | | |
| ***ASCVD Risk Score*** | | | | | | | |
| ASCVD risk score (%) | Median (Q1-Q3) | 4.4 (2.5-6.6) | 3.2 (1.7-5.1) | 4.6 (2.7-6.5) | 5.2 (3.6-7.3) | 6.1 (3.8-8.9) | <0.0001 (0.35) |
|  | 0-<2.5 | 167 (24%) | 101 (37%) | 28 (20%) | 21 (15%) | 17 (12%) |  |
|  | 2.5-<5 | 237 (33%) | 99 (36%) | 52 (36%) | 45 (31%) | 41 (28%) |  |
|  | 5+ | 304 (43%) | 76 (28%) | 63 (44%) | 77 (54%) | 88 (60%) |  |
|  | | | | | | | |
| Smoking status | Current | 166 (24%) | 69 (25%) | 30 (21%) | 38 (27%) | 29 (20%) | 0.470 |
|  | Former | 228 (32%) | 82 (30%) | 43 (30%) | 37 (26%) | 66 (45%) |  |
|  | Never | 312 (44%) | 125 (45%) | 69 (49%) | 67 (47%) | 51 (35%) |  |
|  | | | | | | | |
| Use of antihypertensive medication |  | 138 (19%) | 36 (13%) | 23 (16%) | 36 (25%) | 43 (29%) | 0.090 |
|  | | | | | | | |
| Systolic BP (mmHg) | Median (Q1-Q3) | 122 (113-131) | 120 (112-130) | 122 (114-130) | 123 (115-132) | 123 (116-133) | 0.027 (0.08) |
|  | | | | | | | |
| Total cholesterol (mg/dL) | Median (Q1-Q3) | 186 (162-207) | 185 (160-208) | 187 (169-210) | 188 (165-206) | 180 (149-203) | 0.110 (-0.06) |
|  | | | | | | | |
| HDL-C (mg/dL) | Median (Q1-Q3) | 49 (40-61) | 49 (40-62) | 50 (42-59) | 48 (39-59) | 48 (39-62) | 0.350 (-0.04) |
|  | | | | | | | |
| LDL calculated (mg/dL) | Median (Q1-Q3) | 108 (87-126) | 106 (88-127) | 112 (93-129) | 108 (87-125) | 105 (82-122) | 0.100 (-0.06) |
|  | | | | | | | |
| Triglycerides (mg/dL) | Median (Q1-Q3) | 111 (78-166) | 112 (78-164) | 109 (76-161) | 119 (84-171) | 107 (75-165) | 0.790 (0.01) |
|  | | | | | | | |
|  | | | | | | | |
| ***Biomarkers*** | | | | | | | |
| MCP-1 (pg/mL) | Median (Q1-Q3) | 183.2 (144.9-236.8) | 178.4 (140.8-229.6) | 188.4 (142.1-234.6) | 182.4 (149.4-257.0) | 192.0 (150.0-250.8) | 0.023 (0.09) |
|  | 10%-90% | 117.4-308.1 | 114.8-308.1 | 119.5-282.5 | 117.8-310.6 | 123.2-310.5 |  |
|  | | | | | | | |
| IL-6 (pg/mL) | Median (Q1-Q3) | 1.6 (1.0-2.8) | 1.5 (0.9-2.7) | 1.6 (1.1-2.8) | 1.7 (1.1-2.8) | 1.6 (1.0-3.2) | 0.028 (0.08) |
|  | 10%-90% | 0.8-5.3 | 0.7-4.9 | 0.8-5.3 | 0.8-5.8 | 0.8-8.1 |  |
|  | | | | | | | |
| hs-CRP (mg/L)^3^ | Median (Q1-Q3) | 1.8 (0.8-3.7) | 1.8 (0.8-3.9) | 1.6 (0.8-2.9) | 1.6 (0.9-3.4) | 2.2 (0.9-4.2) | 0.530 (0.02) |
|  | 10%-90% | 0.5-8.6 | 0.4-9.9 | 0.5-6.6 | 0.5-7.0 | 0.5-9.6 |  |
|  | | | | | | | |
| Lp-PLA2 (ng/mL) | Median (Q1-Q3) | 129.1 (91.3-168.2) | 130.4 (90.0-156.4) | 136.9 (97.1-176.1) | 130.7 (97.1-162.4) | 123.2 (88.0-168.2) | 0.780 (0.01) |
|  | 10%-90% | 62.5-207.5 | 58.8-197.4 | 68.8-221.9 | 61.1-207.2 | 69.1-207.5 |  |
|  | | | | | | | |
| oxLDL (U/L) | Median (Q1-Q3) | 53.0 (41.9-69.4) | 54.0 (41.8-66.8) | 53.6 (41.2-68.7) | 50.9 (42.7-74.5) | 52.5 (42.2-66.0) | 0.800 (-0.01) |
|  | 10%-90% | 34.3-87.5 | 34.6-84.3 | 32.4-91.9 | 34.9-87.5 | 33.5-89.2 |  |
|  | | | | | | | |
|  | | | | | | | |
| ***HIV-Related Health Status*** | | | | | | | |
| Duration of HIV (years) | <5 | 66 (9%) | 29 (11%) | 14 (10%) | 14 (10%) | 9 (6%) | 0.110 |
|  | 5-10 | 155 (22%) | 79 (29%) | 34 (24%) | 27 (19%) | 15 (10%) |  |
|  | >10 | 487 (69%) | 168 (61%) | 95 (66%) | 102 (71%) | 122 (84%) |  |
|  | | | | | | | |
| Nadir CD4 (cells/mm³) | <50 | 153 (22%) | 49 (18%) | 28 (20%) | 42 (29%) | 34 (23%) | 0.060 |
|  | 50-199 | 206 (29%) | 77 (28%) | 47 (33%) | 40 (28%) | 42 (29%) |  |
|  | 200-349 | 195 (28%) | 82 (30%) | 42 (29%) | 26 (18%) | 45 (31%) |  |
|  | 350+ | 136 (19%) | 59 (21%) | 24 (17%) | 29 (20%) | 24 (16%) |  |
|  | Unknown | 18 (3%) | 9 (3%) | 2 (1%) | 6 (4%) | 1 (1%) |  |
|  | | | | | | | |
| HIV-1 RNA (copies/mL) | <LLQ | 614 (88%) | 238 (88%) | 125 (88%) | 127 (89%) | 124 (86%) | 0.850 |
|  | LLQ -< 400 | 70 (10%) | 27 (10%) | 15 (11%) | 13 (9%) | 15 (10%) |  |
|  | 400+ | 16 (2%) | 6 (2%) | 2 (1%) | 3 (2%) | 5 (3%) |  |
|  | | | | | | | |
| CD4 count (cells/mm³) | <350 | 106 (15%) | 37 (13%) | 30 (21%) | 18 (13%) | 21 (14%) | 0.280 |
|  | 350-499 | 142 (20%) | 58 (21%) | 27 (19%) | 30 (21%) | 27 (18%) |  |
|  | 500+ | 460 (65%) | 181 (66%) | 86 (60%) | 95 (66%) | 98 (67%) |  |
|  | | | | | | | |
| Total ART use (years) | <5 | 117 (17%) | 54 (20%) | 22 (15%) | 25 (17%) | 16 (11%) | 0.310 |
|  | 5-10 | 184 (26%) | 79 (29%) | 46 (32%) | 29 (20%) | 30 (21%) |  |
|  | 10+ | 407 (57%) | 143 (52%) | 75 (52%) | 89 (62%) | 100 (68%) |  |
|  | | | | | | | |
| ART regimen (by class) | NRTI + INSTI | 309 (44%) | 119 (43%) | 62 (43%) | 72 (50%) | 56 (38%) | 0.750 |
|  | NRTI + NNRTI | 185 (26%) | 84 (30%) | 34 (24%) | 29 (20%) | 38 (26%) |  |
|  | NRTI + PI | 120 (17%) | 42 (15%) | 27 (19%) | 20 (14%) | 31 (21%) |  |
|  | NRTI-sparing | 24 (3%) | 8 (3%) | 6 (4%) | 2 (1%) | 8 (5%) |  |
|  | Other NRTI-containing | 70 (10%) | 23 (8%) | 14 (10%) | 20 (14%) | 13 (9%) |  |
|  | | | | | | | |
|  | | | | | | | |

*^1^All statistics are calculated out of participants with data collected. *P-values for continuous variables are presented as P-value (rho). Categorical variables use Jonckheere-Terpstra. Ordinal variables use Cochran-Mantel-Haenszel with rank scores. ^2^'Other' race/ethnicity includes participants self-identifying as: Asian, Pacific Islander; American Indian, Alaskan Native; native or indigenous to the enrollment region; more than one race (with no single race noted as predominant); or of unknown race. ^3^hs-CRP contains censored values (n=78). Screening lipids were tested locally using various assays and are not necessarily fasting. ASCVD=Atherosclerotic cardiovascular disease; BMI=Body mass index; ART=Antiretroviral therapy; NRTI=Nucleoside/nucleotide reverse transcriptase inhibitor; NNRTI=Non-nucleoside reverse transcriptase inhibitor; PI=Protease inhibitor; INSTI=Integrase-strand transfer inhibitor.*

# Supplemental Table 2. Plaque Features and Cardiovascular Outcomes Across Non-HIV Coronary CT Angiography Studies

| **Plaque Feature** | **CGPS^1^ (n=9533) Adjusted RR (95%CI) (5-year Death/MI)** | **SCOT-HEART^2^ (n=1769)**  **Adjusted HR (95%CI)**  **(4.7-Year Fatal/ Nonfatal MI)** | **PROMISE^3^ (n=4415)**  **Adjusted HR (95% CI) (2.1-Year MACE)** |
| --- | --- | --- | --- |
| Reference | 1.00 (No Subclinical ASCVD) | Per doubling | 1.00 (without  high-risk plaque) |
| Any Subclinical Coronary Atherosclerosis | **1.59 (1.16–2.20)** | NA | NA |
| Nonobstructive Subclinical Coronary Atherosclerosis | **1.44 (1.03–2.00)** | NA | NA |
| Obstructive Subclinical Coronary Atherosclerosis | **2.18 (1.48–3.20)** | NA | NA |
| Multi-VD/LM-Obstructive | **2.61 (1.56–4.36)** | NA | NA |
| Plaque Burden | NA | 1.23 (0.95–1.60) | NA |
| Noncalcified Plaque Burden | NA | 1.21 (0.94–1.56) | NA |
| Low-Attenuation Plaque Burden | NA | **1.60 (1.10–2.34)** | NA |
| Calcified Plaque Burden | NA | 1.42 (0.90–2.25) | NA |
| Any High-Risk Plaque | NA | NA | **1.72 (1.13–2.62)** |
| Both Positive Remodeling and Low CT Attenuation | NA | NA | **1.56 (0.85–2.84)** |
| At Least One of Positive Remodeling or Low CT Attenuation | NA | NA | **1.74 (1.14–2.65)** |
| Abbreviations. ASCVD, atherosclerotic cardiovascular disease; CGPS, Copenhagen General Population Study; CI, confidence interval; HR, hazard ratio; MACE; major adverse event (death from any cause, myocardial infarction, and hospitalization for unstable angina); MI, myocardial infarction; PROMISE, Prospective Multicenter Imaging Study for Evaluation of Chest Pain; SCOT-HEART, Scottish Computed Tomography of the HEART | | | |

**Supplemental Figure 1: Distribution of hs-cTnT Levels by Coronary Plaque Features**

| **A. Plaque Present** | **B. Vulnerable Plaque Present*** |
| --- | --- |
| 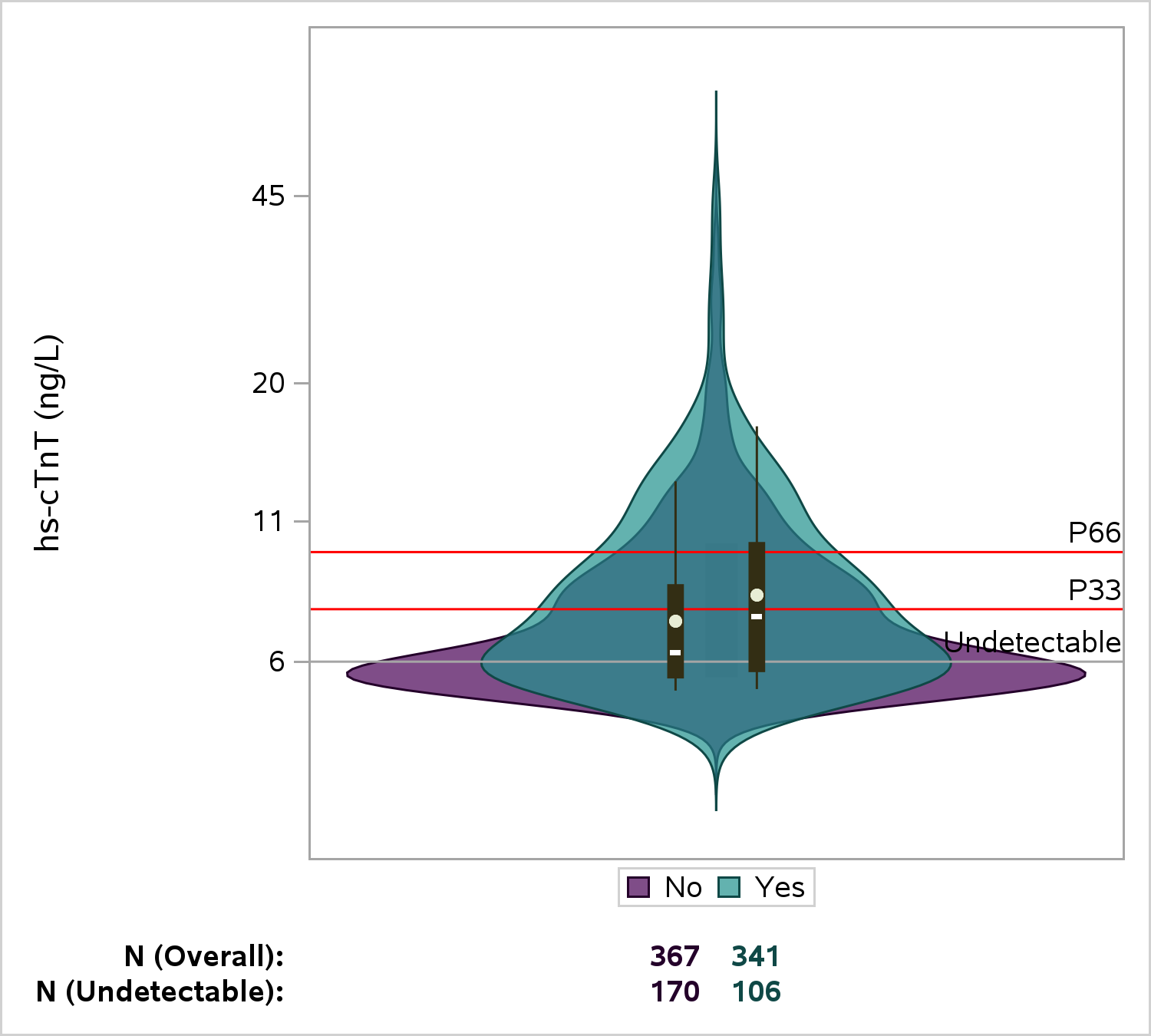 | 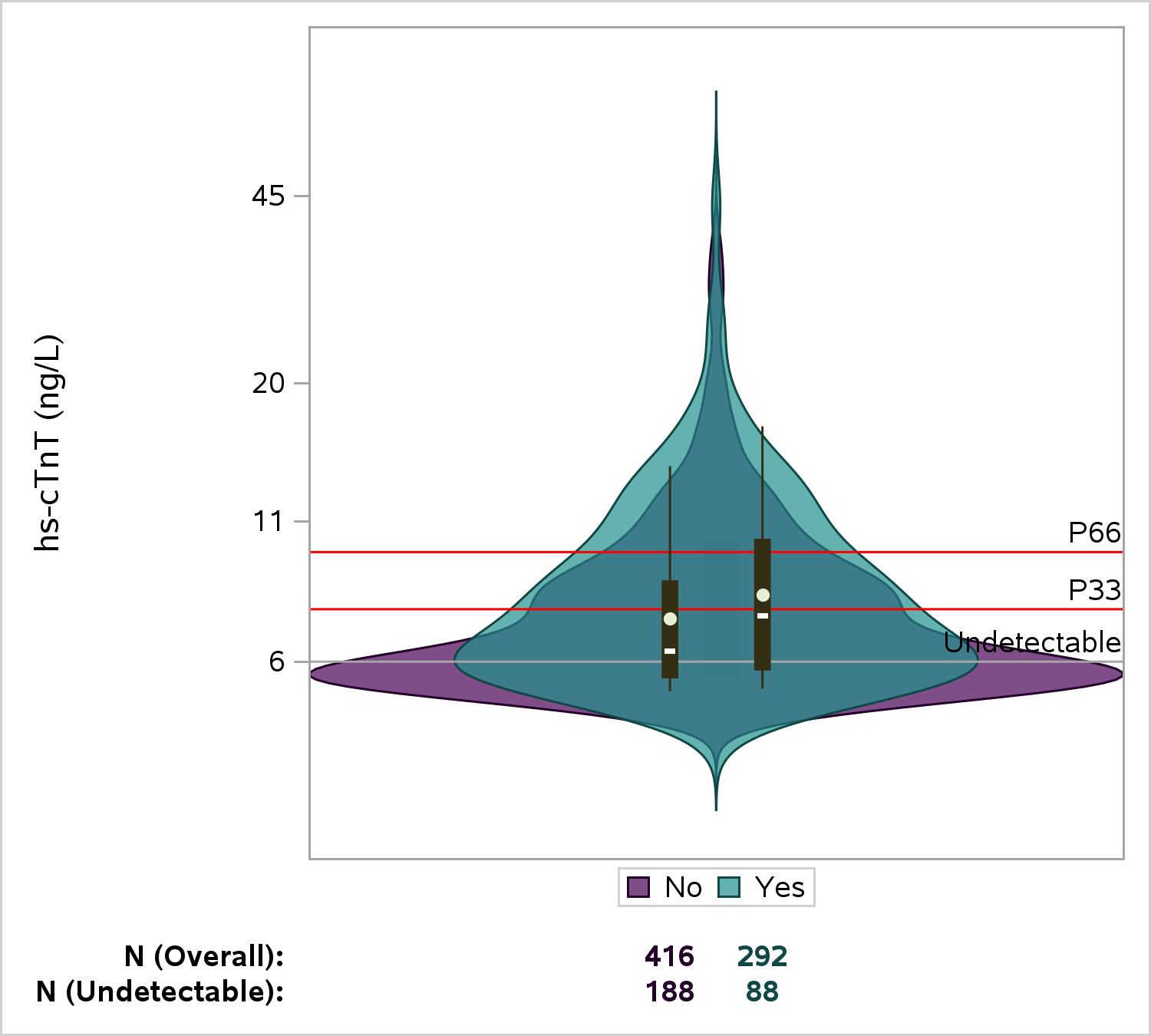 |

| **C. Calcium Score (Agatston)** | **D. Leaman Score** |
| --- | --- |
| 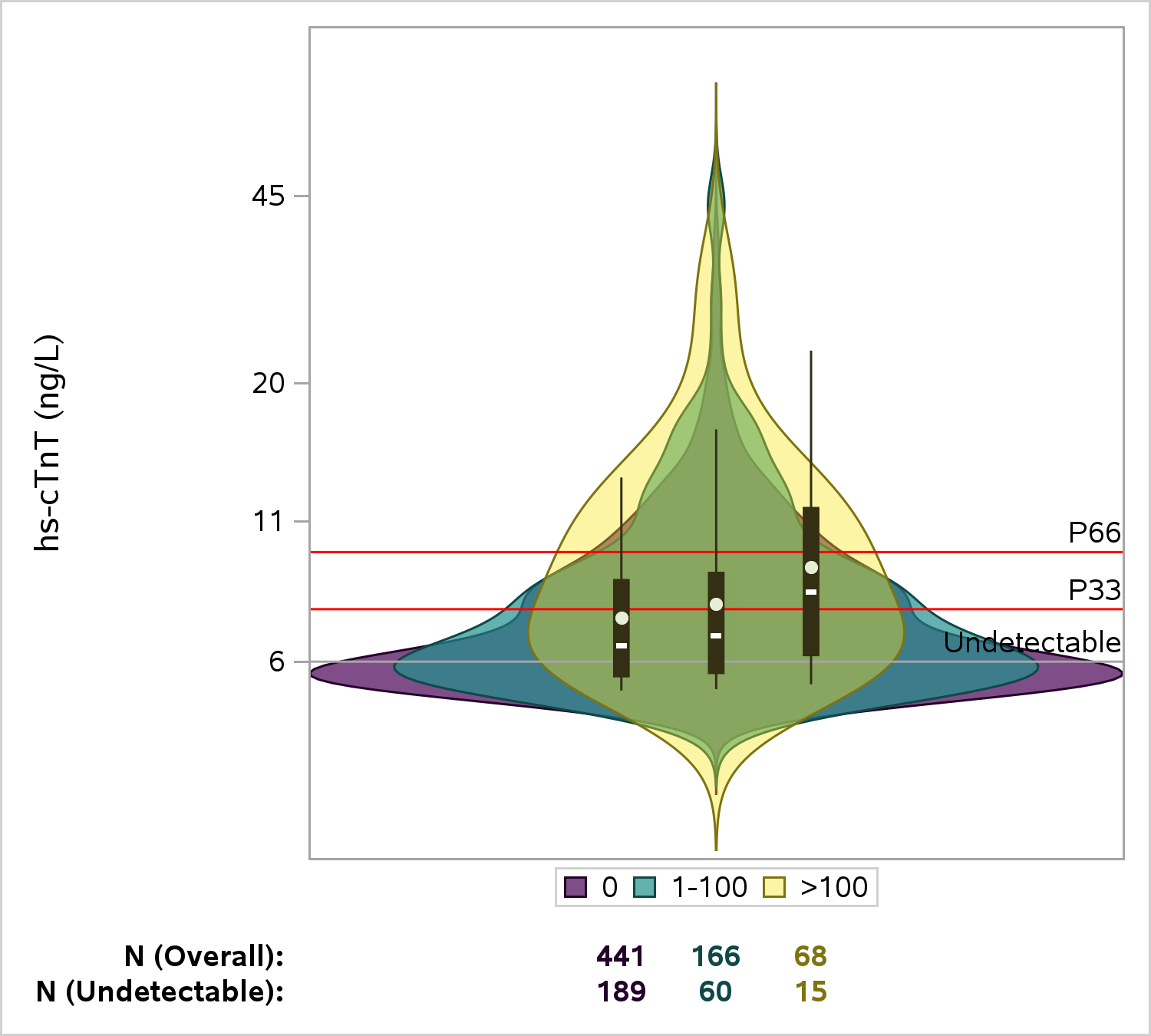 | 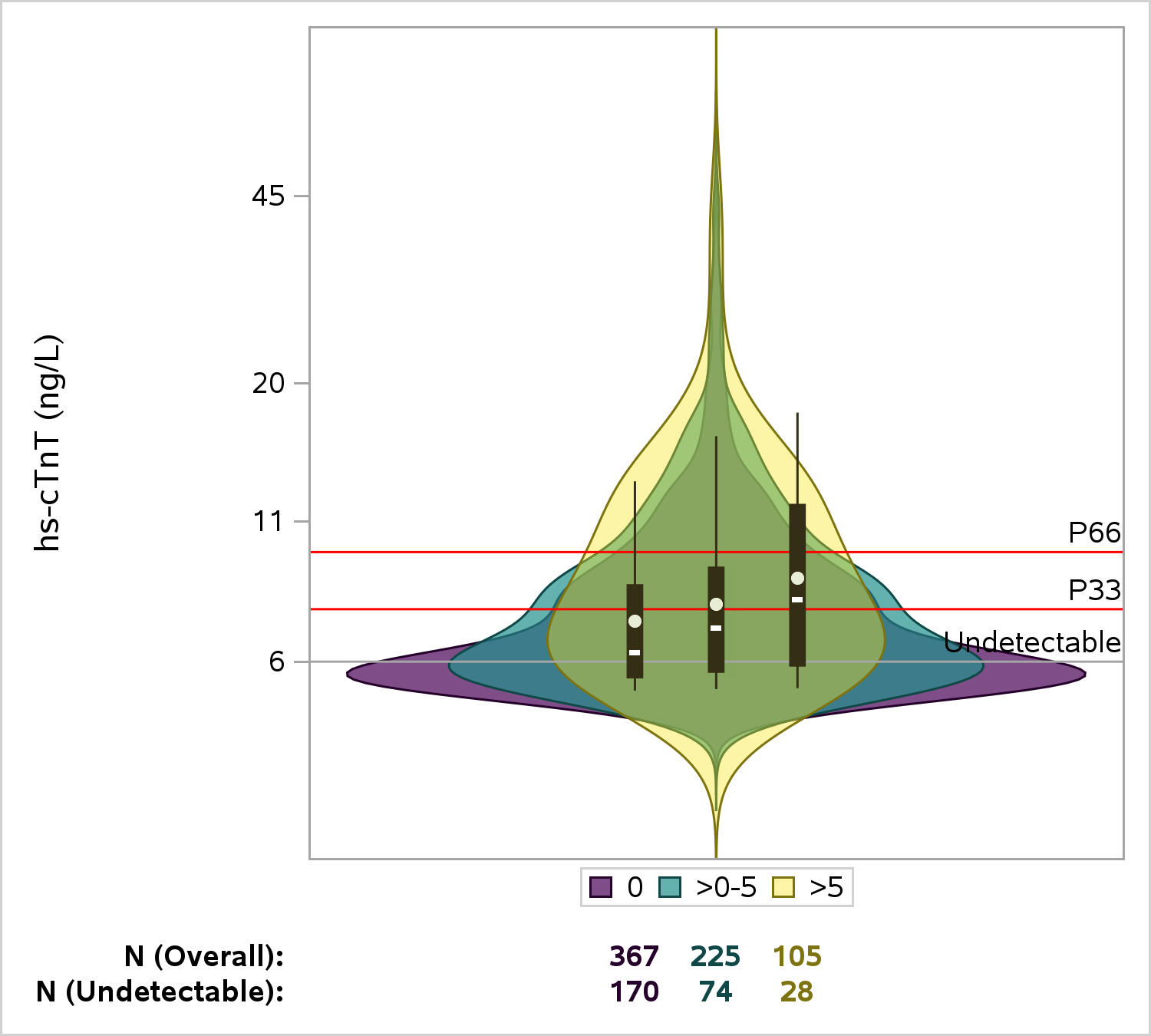 |

**Plaque with Visible Noncalcified Portion or Plaque with Vulnerable Features.*

*Violin plots presenting Kernel estimate of probability density function, and mean (circle), median (white dash), Q1-Q3 (box), P5-P95 (whiskers).*

**Supplemental Figure 2. Association of Prevalence of Plaque Outcomes with hs-cTnT Evaluated as a Continuous Variable Using a Cubic Spline Model and Categorically**


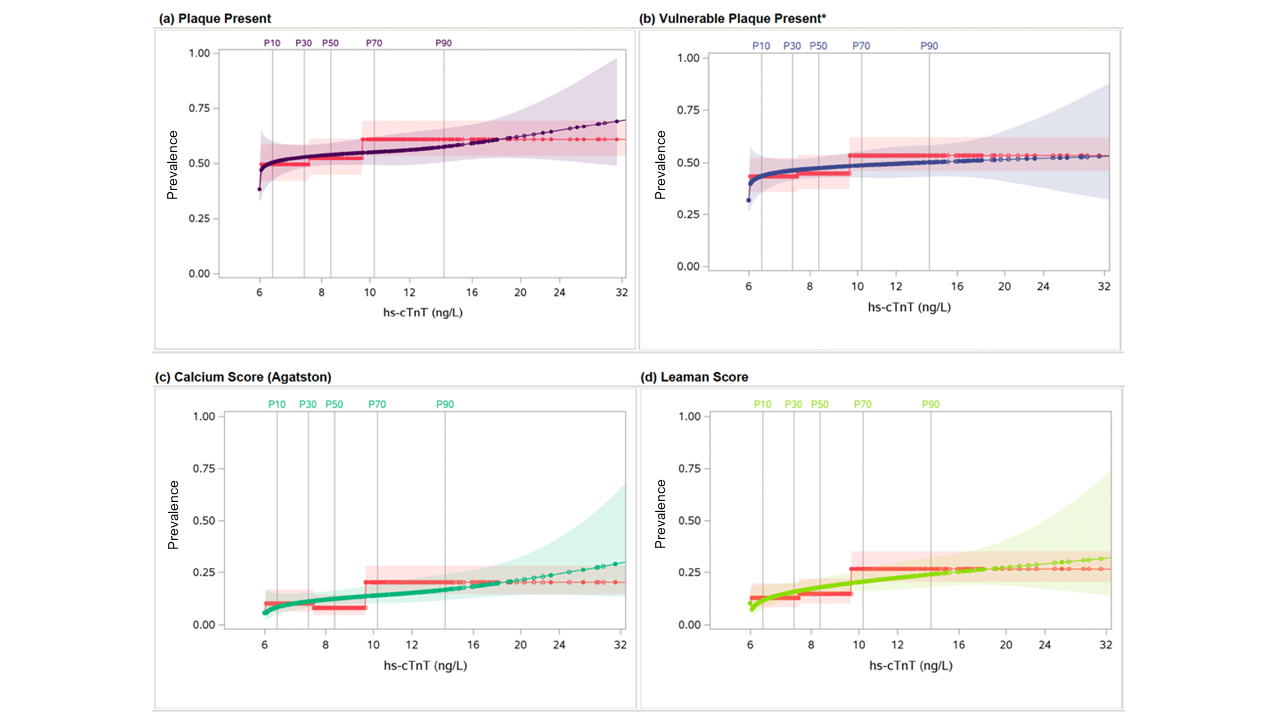


Model includes knots at the 10^th^, 50^th^, 90^th^ percentiles of measurable values. Predicted values and associated 95% confidence bands for the fitted splines are shown over the predicted values for the original categorical model (shown in red). * Plaque with visible noncalcified portion or plaque with vulnerable features.

**Supplemental Figure 3: Log Binomial Regression on Binary Plaque Outcomes by hs-cTnT Level Adjusted for (A.) ASCVD Risk Score + Biomarkers of Inflammation, (B.) ASCVD Risk Score + Biomarkers of Inflammation + Nadir CD4 Count**

| 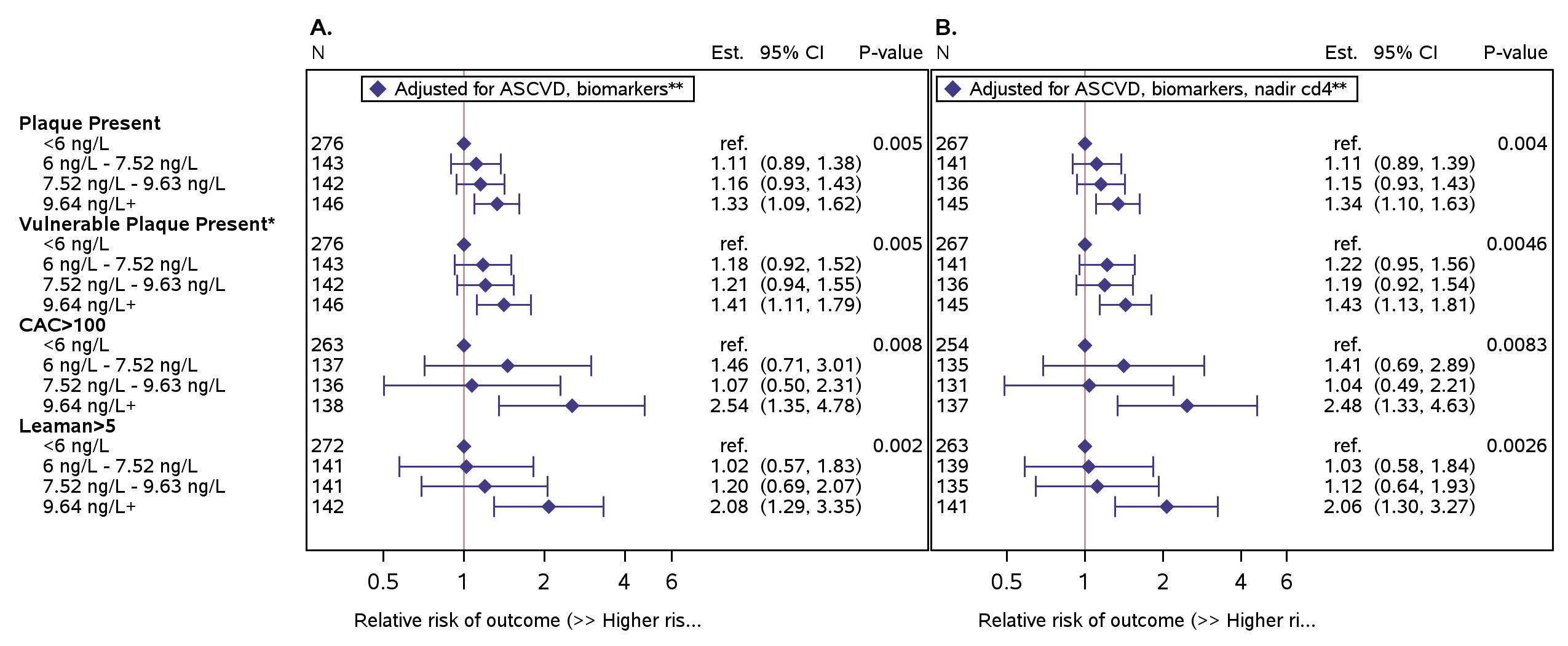 |
| --- |

**Plaque with Visible Noncalcified Portion or Plaque with Vulnerable Features*

***Some of these models did not converge well, we managed to get convergence by dropping biomarkers that had no effect.*

*The p-value tests for a linearly increasing log RR.*

**Supplemental Figure 4: Logistic Regression Modeling for Binary Plaque Outcomes by hs-cTnT Level for (A.) Unadjusted, and (B.) Adjusted for the Pooled Cohort 10-year ASCVD Risk Score**


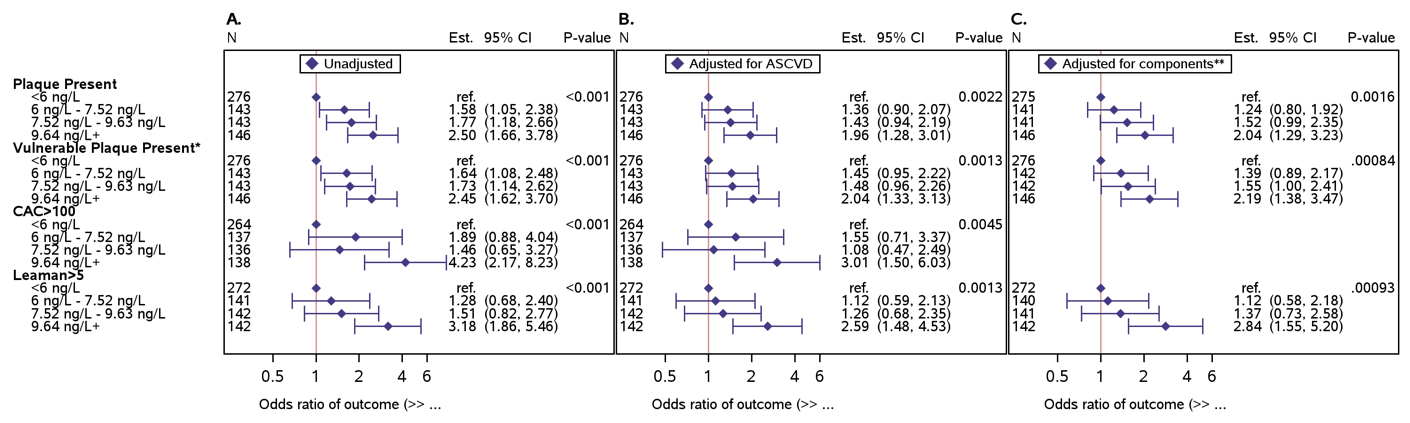


**References**

1. Fuchs A, Kühl JT, Sigvardsen PE et al. Subclinical Coronary Atherosclerosis and Risk for Myocardial Infarction in a Danish Cohort : A Prospective Observational Cohort Study. Ann Intern Med 2023;176:433-442.

2. Williams MC, Kwiecinski J, Doris M et al. Low-Attenuation Noncalcified Plaque on Coronary Computed Tomography Angiography Predicts Myocardial Infarction. Circulation 2020;141:1452-1462.

3. Ferencik M, Mayrhofer T, Bittner DO et al. Use of High-Risk Coronary Atherosclerotic Plaque Detection for Risk Stratification of Patients With Stable Chest Pain: A Secondary Analysis of the PROMISE Randomized Clinical Trial. JAMA cardiology 2018;3:144-152.
